# *Candida albicans* SR-Like Protein Kinases Regulate Different Cellular Processes: Sky1 Is Involved in Control of Ion Homeostasis, While Sky2 Is Important for Dipeptide Utilization

**DOI:** 10.3389/fcimb.2022.850531

**Published:** 2022-05-06

**Authors:** Philipp Brandt, Franziska Gerwien, Lysett Wagner, Thomas Krüger, Bernardo Ramírez-Zavala, Mohammad H. Mirhakkak, Sascha Schäuble, Olaf Kniemeyer, Gianni Panagiotou, Axel A. Brakhage, Joachim Morschhäuser, Slavena Vylkova

**Affiliations:** ^1^ Septomics Research Center, Friedrich Schiller University and Leibniz Institute for Natural Product Research and Infection Biology – Hans Knöll Institute, Jena, Germany; ^2^ Molecular and Applied Microbiology, Leibniz Institute for Natural Product Research and Infection Biology – Hans Knöll Institute, Jena, Germany; ^3^ Institute for Molecular Infection Biology, University of Würzburg, Würzburg, Germany; ^4^ Systems Biology and Bioinformatics, Leibniz Institute for Natural Product Research and Infection Biology – Hans Knöll Institute, Jena, Germany; ^5^ Department of Medicine and State Key Laboratory of Pharmaceutical Biotechnology, University of Hong Kong, Hong Kong, Hong Kong SAR, China

**Keywords:** Sky1, Sky2, dipeptide transport, ion homeostasis, *Candida albicans*, protein kinases

## Abstract

Protein kinases play a crucial role in regulating cellular processes such as growth, proliferation, environmental adaptation and stress responses. Serine-arginine (SR) protein kinases are highly conserved in eukaryotes and regulate fundamental processes such as constitutive and alternative splicing, mRNA processing and ion homeostasis. The *Candida albicans* genome encodes two (Sky1, Sky2) and the *Candida glabrata* genome has one homolog (Sky1) of the human SR protein kinase 1, but their functions have not yet been investigated. We used deletion strains of the corresponding genes in both fungi to study their cellular functions. *C. glabrata* and *C. albicans* strains lacking *SKY1* exhibited higher resistance to osmotic stress and toxic polyamine concentrations, similar to *Saccharomyces cerevisiae sky1*Δ mutants. Deletion of *SKY2* in *C. albicans* resulted in impaired utilization of various dipeptides as the sole nitrogen source. Subsequent phosphoproteomic analysis identified the di- and tripeptide transporter Ptr22 as a potential Sky2 substrate. Sky2 seems to be involved in Ptr22 regulation since overexpression of *PTR22* in the *sky2*Δ mutant restored the ability to grow on dipeptides and made the cells more susceptible to the dipeptide antifungals Polyoxin D and Nikkomycin Z. Altogether, our results demonstrate that *C. albicans* and *C. glabrata* Sky1 protein kinases are functionally similar to Sky1 in *S. cerevisiae*, whereas *C. albicans* Sky2, a unique kinase of the CTG clade, likely regulates dipeptide uptake *via* Ptr22.

## Introduction

The human fungal pathogen *Candida albicans* colonizes various host niches, such as the oral cavity, the gastrointestinal and urogenital tracts, and the skin of healthy individuals. In immunocompromised patients and other susceptible individuals it can cause mucosal or systemic infections (Odds, 1988). Both its commensal and pathogenic lifestyles are regulated *via* complex networks in which protein kinases play an essential role. The *C. albicans* genome comprises 108 predicted protein kinases, many of which were shown to regulate cellular growth and proliferation, resistance to environmental stresses, and the expression of virulence attributes (Monge et al., 2006; Hogan and Sundstrom, 2009; Ramírez-Zavala et al., 2017). Despite their importance for commensalism and pathogenicity, more than half of the protein kinases in *C. albicans* have not yet been characterised in detail. In a previous work, Ramírez-Zavala et al. generated a mutant library of 17 non-essential uncharacterized predicted protein kinases without an assigned gene name and identified orf19.3840 as a crucial activator of the protein kinase Snf1 in *C. albicans* (Ramírez-Zavala et al., 2017). In this study, we focus on the function of other uncharacterized predicted protein kinases in *C. albicans*, specifically Sky1 (CaSky1) and orf19.35 (CaSky2), homologs of the human serine-arginine protein kinase (SRPK) SRPK1.

The SRPK subfamily is highly conserved from yeast to humans. They commonly catalyze the phosphorylation of mRNA regulatory proteins enriched in serine/arginine recognition motifs and therefore termed SR proteins (Zahler et al., 1992). Human SRPK1–3 are critical for the regulation of both constitutive and alternative splicing, mRNA nuclear export and stability, as well as translational control *via* shuttling of SR proteins to the cytoplasm (Zhou and Fu, 2013). In addition, human SRPK1 is also exploited during viral infections to facilitate the viral cell cycle and can also act as a tumor suppressor by modulating the state of the Akt kinase, a hallmark of several cancers like prostate, breast, and lung cancer (Hayes et al., 2007; Karakama et al., 2010; Odunsi et al., 2012; Wang et al., 2014).

Fungal SRPK-like kinases can be found across the whole fungal kingdom, including the phyla Ascomycota, Basidiomycota, Mucormycota and Zoopagomycota. However, their functions were studied only in the fungal plant pathogens *Fusarium graminearum*, *Physarum polycephalum*, and *Puccinia striiformis f.* sp. *Tritici* (Liu et al., 2009; Cheng et al., 2015; Wang et al., 2018) and in the model yeasts *Saccharomyces cerevisiae* and *Schizosaccharomyces pombe*. Both model yeasts have only a single SRPK-like protein: Dsk1 in *S.pombe* regulates mitosis and pre-mRNA splicing (Takeuchi and Yanagida, 1993; Tang et al., 1998; Tang et al., 2007), whereas Sky1 in *S. cerevisiae* (ScSky1) broadly regulates cellular functions. For example, ScSky1 phosphorylates Npl3, an SR-like protein involved in histone H2B ubiquitination, pre-mRNA splicing, and export of mRNA from the nucleus (Moehle et al., 2012). Further, ScSky1 regulates polyamine transport and is involved in ion homeostasis and salt tolerance (Erez and Kahana, 2001; Forment et al., 2002).

The genome of most common pathogenic *Candida* spp. including *C. albicans*, *Candida auris*, *Candida parapsilosis* and *Candida tropicalis* encodes two predicted SR-like protein kinases, except for *Candida glabrata* which, similarly to *S. cerevisiae*, possesses only one predicted SRPK member (CgSky1). We deleted *SKY1* in *C. glabrata* and included this mutant strain as well as the available Sc*sky1*Δ, Ca*sky1*Δ and Ca*sky2*Δ (orf19.35) mutants in our study in order to gain insight into the conservation of the regulated mechanisms. We tested the mutant strains for resistance to osmotic stress and toxic polyamine concentrations, as previously reported for the Sc*sky1*Δ mutant. Mutants lacking *SKY1* (Sc*sky1*Δ, Cg*sky1*Δ and Ca*sky1*Δ) were more resistant to these stressors compared to the respective wild-type strains, which indicates a conserved functional similarity of Sky1. Phosphoproteomic analysis of Ca*sky1*Δ and Ca*sky2*Δ mutants revealed little overlap in the potential phosphorylation target proteins of these two kinases, further highlighting the functional differences between CaSky1 and CaSky2 in *C. albicans*. One potential phosphorylation target of CaSky1 is the uncharacterized protein kinase Hrk1, which shared the Sky1 function in regulation of ion homeostasis. Among the candidate target proteins of CaSky2 was Ptr22, a di- and tripeptide transporter in *C. albicans*. Functional analysis confirmed that CaSky2 is important for dipeptide utilization, a novel role for SR kinases. Overexpression of *PTR22* in the Ca*sky2*Δ mutant restored the ability to grow on dipeptides as sole nitrogen source and rendered the cells more susceptible to the dipeptide antifungals Polyoxin D and Nikkomycin Z. Altogether, our results demonstrate the distinct functions of the two SR-like protein kinases in *C. albicans*, with Sky1 regulating ion homeostasis and CaSky2 likely being involved in regulation of dipeptide uptake *via* the Ptr22 transporter.

## Materials and Methods

### Strains and Growth Conditions

The *C. albicans*, *C. glabrata*, and *S. cerevisiae* strains used in this study are listed in **Supplementary Table 1**. All strains were stored as frozen stocks containing 20% glycerol at -80°C and sub-cultured on YPD agar plates (1% yeast extract, 2% peptone, 2% glucose, 2% agar) at 30°C for 2 days. Strains were routinely grown in YPD liquid medium at 30°C overnight with shaking at 180 rpm.

### *Candida albicans* Strain Construction

*C. albicans Casky1*Δ and*Casky2*Δ (orf19.35) deletion mutants were taken from the existent mutant library of non-essential uncharacterized predicted protein kinases (Ramírez-Zavala et al., 2022). All other *C. albicans* deletion mutants and complemented strains were constructed as described previously (Ramírez-Zavala et al., 2017). *C. albicans PTR22* overexpression strains were generated using the ApaI-SacII fragment from plasmid pPTR22E1 to integrate *PTR22* under the control of the *ADH1* promoter in the wild-type strain SC5314 and the *sky2*Δ mutants as described previously (Reuss et al., 2004; Dunkel et al., 2013). The correct genomic integration of all constructs was confirmed by Southern hybridization with the upstream and downstream flanking sequences. All strains and primers are listed in **Supplementary Table 1**.

### *Candida glabrata* Strain Construction

The generation of Cg*sky1*Δ was conducted with a PCR-based Gibson Assembly cloning approach (NEB) according to the manufacturers’ protocol. Purified PCR fragments for the puC19 vector backbone, the Cg*SKY1* 5’ flank (~1000 bp), a barcoded nourseothricin resistance cassette (*NAT1*) with constant flanking regions (U1 and D1) derived from the mutant 7G6 from Schwarzmüller et al. (2014), and the Cg*SKY1* 3’ flank (~1000 bp) were fused into one vector. The deletion construct was verified by sequencing, then PCR-amplified and used to transform the *C. glabrata* wild-type strain ATCC2001 by a modified heat shock method (with 45°C heat shock for 15 min) (Sanglard et al., 1996). The transformants were plated onto YPD agar plates containing 200 µg/ml nourseothricin and positive knockout strains were verified by control PCRs. All strains and primers are listed in **Supplementary Table 1**.

### *Saccharomyces cerevisiae* Strain Construction

The *S. cerevisiae* wild-type strain BY4741 (Y00000) and the Sc*sky1*Δ mutant (YMR216C) were obtained from Euroscarf (www.euroscarf.de). Both strains were transformed with the plasmid pHLUM (Addgene, Massachusetts, USA) by a LiAc/SS carrier DNA/PEG method to restore the non-auxotrophic strain background as described previously (Gietz and Schiestl, 2007).

### Multiple Alignment Analysis

**Figure 1:**
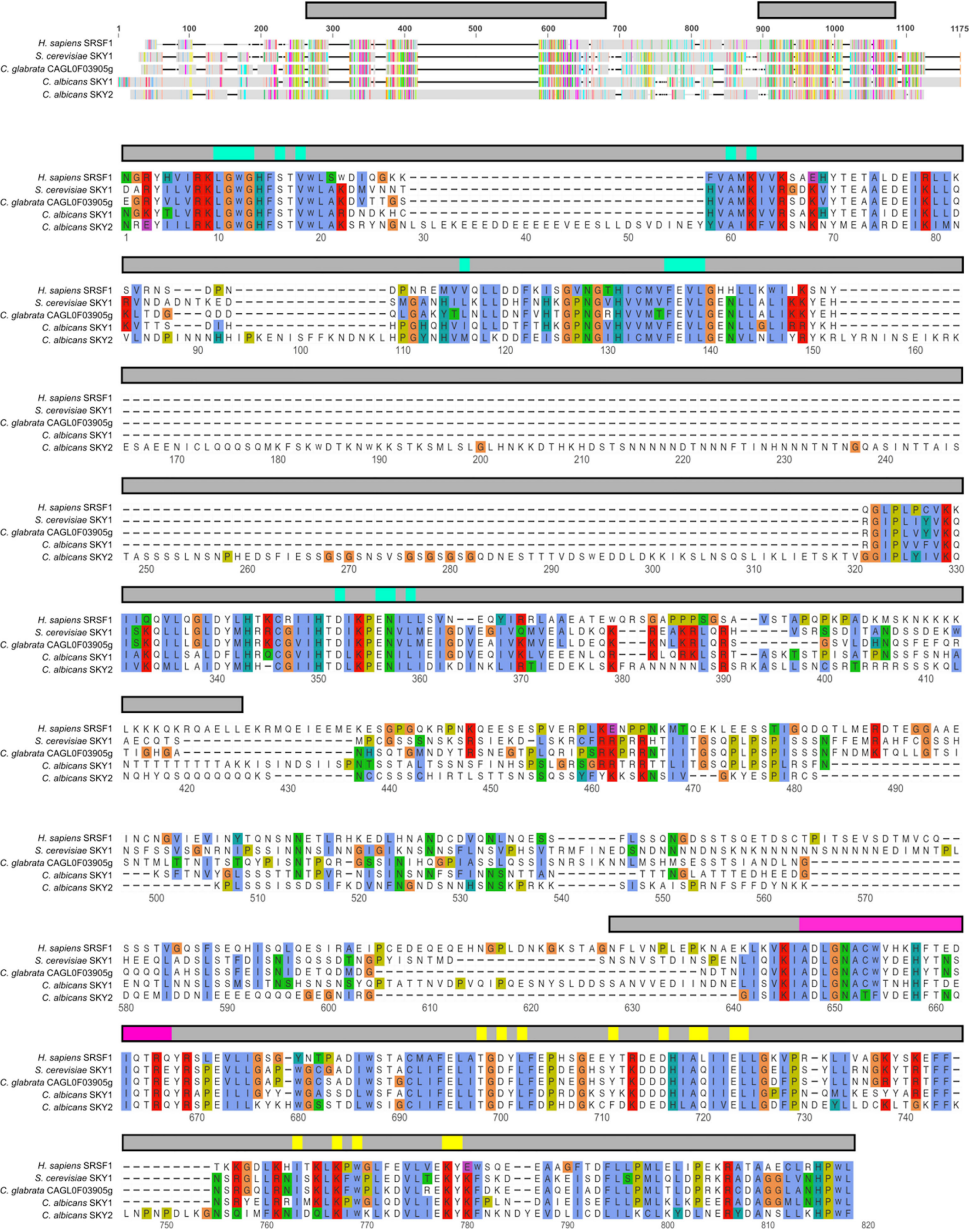
Protein alignment of SRPK homologs: *H. sapiens* SRPK1, *S. cerevisiae* Sky1, *C. glabrata* Sky1, and *C. albicans* Sky1 and Sky2. The annotation of the protein features (the bars above the corresponding alignment section) is based on the annotation of the human SRPK 1 (NP_003128.3). Upper graph: Overview alignment of the entire protein, where the kinase domains are indicated by the grey bars above. Lower graph: Detailed alignment of the kinase domain (grey bar above) intercepted by the ‘spacer’ region. The protein kinase domain contains ATP binding sites (turquoise), activation loop (magenta) and kinase-docking sites (yellow). All regions required for SRPK1 function are highly conserved among the fungal species. The conserved alignment sites are highlighted within the alignment applying Clustal X default coloring.

The alignment was generated using ‘MUSCLE Alignment’ implemented in Geneious Prime (v2020.1.1.) using default settings. Sequence data originates from GenBank (NP_003128, NP_013943) and the *Candida* Genome Database (C1_06090C, C2_06600W, CAGL0F03905g). The data presented in **Figure 1** was exported from Geneious Prime. The detailed alignment of the kinase domain is illustrated by applying the R-package ‘ggmsa’ (by Guangchuang Yu, https://CRAN.R-project.org/package=ggmsa) using the color scheme ‘Clustal’.

### High-Throughput Phenotypic Screen

The *C. albicans* wild-type strain SC5314 and strain A of the Ca*sky1*Δ and Ca*sky2*Δ mutants (**Supplementary Table 1**) were pre-grown on YPD plates. High-throughput phenotypic screen was performed using Phenotype MicroArrays for microbial cells (PM) plates, reagents, and devices according to the manufacturers’ instructions (Biolog, Inc., USA). Briefly, *C. albicans* cells were scraped from YPD agar plates and the cell number was adjusted in sterile dH_2_O to 62% transmittance as measured by a turbidimeter (Biolog, Inc., USA). The cells were added to inoculating fluid IFY-0 base (1.2x), redox dye mix D (75x) (Biolog Inc., USA), and further supplemented with potassium phosphate, sodium sulfate and either glucose or glutamic acid (Sigma-Aldrich). 100 µl of the respective mixture was added to each well of PM plates for fungi (PM1–10 and PM21–25) to test for metabolic activity in the presence of different carbon sources, nitrogen sources, supplements, and chemicals. Plates were incubated at 37°C in an OmniLog multiple plate reader (Biolog, Inc., USA). Metabolic activity was determined by a colorimetric reduction of the redox dye and kinetically measured every 15 min at an optical density OD of 750 nm for 24–48 h. Each experiment was performed in duplicates.

For analysis, the respective negative control was substracted from the growth signals in each Phenotype MicroArray. Negative values were replaced by zero, if present. Next, each array’s growth signals were categorized into two groups: exponential growth (active growth) and no exponential growth (non-active growth), as proposed by Vehkala et al. (2015). In brief, a data fitted logistic curve represented exponential growth, while a line without showing exponential growth phase characteristics was interpreted as non-active growth in the investigated time frame. The method was repeated for each replicate separately. Hence, a given substrate’s growth signal was detected as exponential growth in two replicates or in no replicates if the analysis identified the same behavior for both replicates. Expoential growth in one replicate was identified if not all replicates for a given tested metabolite showed active growth. Due to low sample numbers, growth differences were assessed by comparing log_2_(fold changes) of the last time point per substrate per phenotypic microarray assay. Grouping the growth signals was implemented in R version 3.6.0 with the pipeline proposed by Vehkala et al., (Vehkala et al., 2015) built upon the opm package version 1.3.77 (Vaas et al., 2013).

### Growth Assays

YPD overnight cultures of the wild type and mutant strains were centrifuged (4,000 × g, 5 min) and washed twice with dH_2_O. Susceptibility to osmotic stress and stress caused by polyamines was tested by spot dilution assays on YPD agar plates as described in the respective figure legends. Briefly, strains were adjusted to an optical density of 600 nm (OD_600_) of 1.0 and 5 µl of each 10-fold serial dilutions was spotted onto the respective plates and incubated for 2 days at either 30°C or 37°C.

Growth curves in liquid media were performed in YCB medium (11.7 g/l yeast carbon base, pH 5.0) containing 10 mM of the respective dipeptides. YPD overnight cultures of the respective *C. albicans* strains were centrifuged (4,000 × g, 5 min) and washed twice with dH_2_O. Strains were adjusted to an OD_600_ of 0.01 in 12 ml YCB medium containing 10 mM of the respective dipeptides and incubated for 48 h at 37°C shaking at 180 rpm. The OD_600_ was measured after 24 h and 48 h. In addition, growth curves were performed in SD medium (0.17% (w/v) yeast nitrogen base without ammonium sulfate and amino acids, 0.5% (w/v) ammonium sulfate, 2% (w/v) glucose, pH 5.0) supplemented with 140 µg/ml Nikkomycin Z (Sigma-Aldrich) or 130 µg/ml Polyoxin D (Sigma-Aldrich) to test for sensitivity to these antibiotics. Strains were adjusted to an OD_600_ of 0.02 in 100 µl of the respective medium in 96-well plates. Plates were incubated at 37°C in an Infinite 200 Pro plate reader (Tecan, Germany) and absorbance was measured every 20 min at an optical density of 600 nm for 24 h or 48 h. Each strain was measured in triplicates and the means and standard deviations were calculated.

### Proteomics Sample Preparation

YPD overnight cultures of the wild-type strain SC5314 and mutant strains were adjusted to an OD_600_ of 0.1 in 900 ml YPD medium and grown for 4 h at 37°C. Cells were disrupted by using mortar and pestle with liquid nitrogen. Cell debris were homogenized in lysis buffer (1% (w/v) SDS, 150 mM NaCl, 100 mM TEAB (triethyl ammonium bicarbonate), one tablet each of cOmplete Ultra Protease Inhibitor Cocktail and PhosSTOP). After addition of 0.5 µl Benzonase nuclease (250 U/μl) the samples were incubated at 37°C in a water bath sonicator for 30 min. Proteins were separated from insolubilized debris by centrifugation (15 min, 18000 × *g*). Each 6 mg of total protein per sample was diluted with 100 mM TEAB to gain a final volume of 4 ml. Subsequently, cysteine thiols were reduced and carbamidomethylated in one step for 30 min at 70°C by addition of 120 µl of 500 mM TCEP (tris(2-carboxyethyl)phosphine) and 120 µl of 625 mM 2-chloroacetamide (CAA). The samples were further cleaned up by methanol-chloroform-water precipitation using the protocol of Wessel and Flügge (Wessel and Flügge, 1984). Protein precipitates were resolubilized in 5% trifluoroethanol of aqueous 100 mM TEAB and digested overnight (18 h) with a Trypsin+LysC mixture (Promega) at a protein to protease ratio of 33:1. Each sample was divided in 6 × 1 mg used for the phosphopeptide enrichment and 200 µg initial protein used as reference for proteome analysis. Samples were evaporated in a SpeedVac. The reference proteome sample was resolubilized in 50 µl of 0.05% TFA in H_2_O/ACN 98/2 (v/v) filtered through Ultrafree-MC 0.2 µm PTFE membrane spin filters (Merck-Millipore). The filtrate was transferred to HPLC vials and injected into the LC-MS/MS instrument for further analysis.

### Phosphopeptide Enrichment

Phosphopeptides were enriched by using TiO_2_+ZrO_2_ TopTips (Glygen Corp., Columbia, MD, USA). TopTips were loaded with 1 mg protein isolate using 6 TopTips per biological replicate after equilibration with 200 µl Load and Wash Solution 1, LWS1 (1% trifluoroacetic acid (TFA), 20% lactic acid, 25% acetonitrile (ACN), 54% H_2_O). TopTips were centrifuged at 1500 rpm (~200 × *g*) for 5 min at room temperature. After washing with 200 µl LWS1, the TiO_2_/ZrO_2_ resin was washed with 25% ACN and subsequently the phosphopeptides were eluted with 200 µl NH_3_· H_2_O (NH_4_OH), pH 12. The alkaline solution was immediately evaporated using a vacuum concentrator (Eppendorf). The phosphoproteome samples were resolubilized in 50 µl of 0.05% TFA in H_2_O/ACN 98/2 (v/v) filtered through Ultrafree-MC 0.2 µm PTFE membrane spin filters (Merck-Millipore). The filtrate was then transferred to HPLC vials and injected into the LC-MS/MS instrument for further analysis.

### LC-MS/MS Analysis

Each sample was measured in triplicates (3 analytical replicates of 3 biological replicates of a reference proteome fraction and a phosphoproteome fraction). LC-MS/MS analysis was performed on an Ultimate 3000 nano RSLC system connected to a QExactive HF mass spectrometer (Thermo Fisher Scientific, Waltham, MA, USA). Peptide trapping for 5 min on an Acclaim Pep Map 100 column (2 cm × 75 µm, 3 µm) at 5 µl/min was followed by separation on an analytical Acclaim Pep Map RSLC nano column (50 cm × 75 µm, 2 µm). Mobile phase gradient elution of eluent A (0.1% (v/v) formic acid in water) mixed with eluent B (0.1% (v/v) formic acid in 90/10 acetonitrile/water) was performed using the following gradient for the more hydrophilic phosphoproteome samples: 0–5 min at 4% B, 15 min at 7% B, 50 min at 10% B, 100 min at 14% B, 150 min at 25% B, 190 min at 60% B, 205–215 min at 96% B, 215–240 min at 4% B. The reference proteome gradient was as follows: 0–4 min at 4% B, 10 min at 7% B, 50 min at 12% B, 100 min at 16% B, 150 min at 25% B, 175 min at 35% B, 200 min at 60% B, 210–215 min at 96% B, 215–240 min at 4% B.

Positively charged ions were generated at a spray voltage of 2.2 kV using a stainless-steel emitter attached to the Nanospray Flex Ion Source (Thermo Fisher Scientific). The quadrupole/orbitrap instrument was operated in Full MS/data dependent MS2 Top15 mode. Precursor ions were monitored at m/z 300–1500 at a resolution of 120,000 FWHM (full width at half maximum) using a maximum injection time (ITmax) of 120 ms and an AGC (automatic gain control) target of 3 × 10^6^. Precursor ions with a charge state of z=2–5 were filtered at an isolation width of *m/z* 1.6 amu for further HCD fragmentation at 30% normalized collision energy (NCE). MS2 ions were scanned at 15,000 FWHM (ITmax=100 ms, AGC= 2 × 10^5^) using a fixed first mass of *m/z* 120 amu. Dynamic exclusion of precursor ions was set to 30 s and the minimum AGC target for Precursor ions selected for HCD fragmentation was set to 1e3. The LC-MS/MS instrument was run by Chromeleon 7.2, QExactive HF Tune 2.8 and Xcalibur 4.0 software.

### Protein Database Search

Tandem mass spectra were searched against the UniProt database (2021/07/19) (YYYY/MM/DD); (https://www.uniprot.org/proteomes/UP000000559) of *Candida albicans* SC5314 using Proteome Discoverer (PD) 2.4 (Thermo) and the algorithms of Mascot 2.4.1 (Matrix Science, UK), Sequest HT (version of PD2.4), MS Amanda 2.0, and MS Fragger 3.2. Two missed cleavages were allowed for the tryptic digestion. The precursor mass tolerance was set to 10 ppm and the fragment mass tolerance was set to 0.02 Da. Modifications were defined as dynamic Met oxidation, phosphorylation of Ser, Thr, and Tyr, protein N-term acetylation as well as static Cys carbamidomethylation. A strict false discovery rate (FDR) < 1% (peptide and protein level) and a search engine score of > 30 (Mascot), > 4 (Sequest HT), > 300 (MS Amanda) or > 8 (MS Fragger) was required for positive protein hits. The Percolator node of PD2.4 and a reverse decoy database was used for q value validation of spectral matches. Only rank 1 proteins and peptides of the top scored proteins were counted. Label-free protein quantification was based on the Minora algorithm of PD2.4 using the precursor abundance based on intensity and a signal-to-noise ratio > 5. Normalization was performed by using the total peptide amount method. Imputation of missing quan values was applied by using abundance values of 75% of the lowest abundance identified per sample. For the reference proteome analysis used for master protein abundance correction of the phosphoproteome data, phosphopeptides were excluded from quantification. Differential protein abundance was defined as a fold change of > 4, ratio-adjusted p-value < 0.05 (p-value/log_4_ratio) and at least identified in 3 of 3 replicates. Differential phosphopeptide abundance was defined as a fold change of > 4, ratio-adjusted p-value < 0.05 (p-value/log_4_ratio) and at least identified in 2 of 3 replicates. The mass spectrometry proteomics data have been deposited to the ProteomeXchange Consortium *via* the PRIDE (Perez-Riverol et al., 2019) partner repository with the dataset identifier PXD027612.

## Results

### Sky1 and Sky2 Are the Sole Predicted SR-Like Protein Kinases in *C. albicans*

According to the *Candida* Genome Database (CGD), the *C. albicans* genome encodes two predicted SR protein kinases, based on sequence similarity to the sole SR-like protein kinase in *S. cerevisiae*, ScSky1. These orthologs correspond to the proteins encoded by C1_06090C (48.5% identity to ScSky1) and C2_06600W (46.5% identity to ScSky1), named in this study CaSky1 and CaSky2, respectively. To ensure that these are the only SRPKs in the pathogenic fungus *C. albicans* and to identify homologs in other pathogenic *Candida* spp., we performed a protein-protein Basic Local Alignment Search Tool (BLASTp) analysis on the CGD platform with the whole human SR protein kinase 1 protein (SRPK1) (NCBI Reference: NP_003128.3) as query. This verified the annotation in the CGD and revealed no further homologs. Interestingly, except for *C. glabrata*, the most common pathogenic *Candida* spp. possess two SRPK homologs (**Supplementary Figure 1**). The CAGL0F03905g-derived protein in *C. glabrata* CgSky1 has sequence identity of 60.35% to ScSky1 (NCBI BLASTp). This illustrates the phylogenetic relationships between the examined species: *C. glabrata* is more closely related to *S. cerevisiae* compared to the other *Candida* species that belong to the CTG clade (Muñoz et al., 2018). Thus, we decided to include CgSky1 in our further analyses to better understand the degree of evolutionary conservation of SR-kinases in *Candida* spp. Multiple sequence alignment showed that the domains that contain the ATP binding sites, the activation loop and the kinase-docking sites are particularly highly conserved between *Homo sapiens*, *S. cerevisiae*, *C. glabrata*, and *C. albicans* (**Figure 1**). Interestingly, the CaSky2 protein has three insertions of amino acid strings of considerable length which are scattered throughout the kinase domain (alignment position 29–57, 94–108 and 153–320) that are not present in the other proteins (**Figure 1**).

### *SKY1* Deletion in *C. glabrata* and *C. albicans* Results in Higher Resistance to Osmotic and Polyamine Stress

**Figure 2:**
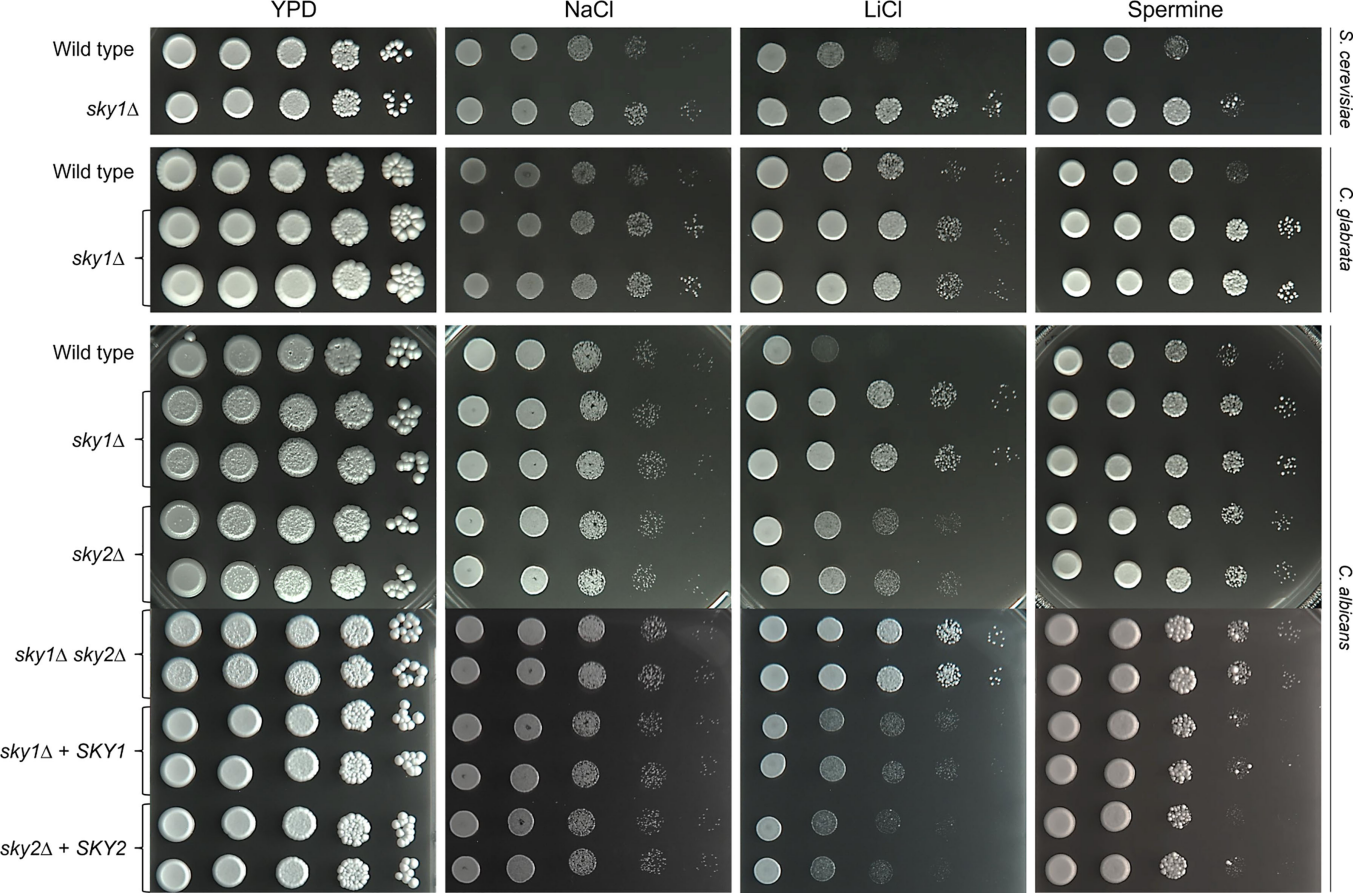
Growth of *S. cerevisiae* and *C. glabrata sky1*Δ mutants and of *C. albicans sky1*Δ and *sky2*Δ mutants on high salt and toxic spermine levels. YPD overnight cultures of the strains were adjusted to an optical density (OD_600_) of 1.0. For *C. albicans* and *C. glabrata* two independently generated mutants were tested. Serial 10-fold dilutions were spotted on YPD agar plates containing the indicated stressor and incubated for 2–3 days at 30°C (*S. cerevisiae*) or 37°C (*C. albicans* and *C. glabrata*). *S. cerevisiae*/*C. glabrata*/*C. albicans* plates contained NaCl (1 M/1.7 M/1.8 M), LiCl (0.3 M/0.18 M/0.5 M) or spermine (2 mM/8 mM/12.8 mM).

*S. cerevisiae* Sky1 has been characterized as a regulator of polyamine transport and ion homeostasis, as mutants lacking *SKY1* are more resistant to toxic cations and polyamine concentrations (Erez and Kahana, 2001). Ion homeostasis is important for *C. albicans* and *C. glabrata* growth and proliferation, survival in the host, virulence mechanisms and resistance to antifungal drugs (Llopis-Torregrosa et al., 2016; Li et al., 2018). To test whether CgSky1, CaSky1, and CaSky2 are involved in similar cellular functions we generated *C. glabrata* mutant strains lacking *SKY1* (Cg*sky1*Δ) and *C. albicans* strains lacking both *SKY1* and *SKY2* (Ca*sky1*Δ Ca*sky2*Δ). In addition, we used available *C. albicans* Ca*sky1*Δ and Ca*sky2*Δ (orf19.35) mutants (Ramírez-Zavala et al., 2022). As a control we used the *S. cerevisiae* BY4741 wild-type strain and a *sky1*Δ mutant (Sc*sky1*Δ). In accordance with previous observations, the Sc*sky1*Δ mutant was more resistant to osmotic stress caused by NaCl or LiCl and to high concentrations of the polyamine spermine (Erez and Kahana, 2001; Forment et al., 2002). Similar phenotypes were observed for *C. glabrata* mutants lacking *SKY1* (**Figure 2**). Deletion of *SKY1* in *C. albicans* resulted in high resistance to LiCl and spermine, whereas lack of *SKY2* rendered the cells partially resistant to these stressors. The Ca*sky1*Δ Ca*sky2*Δ double mutant had the same phenotype as the Ca*sky1*Δ deletion mutant. None of the tested *C. albicans SKY* deletion mutant strains exhibited altered resistance to NaCl, in contrast to the Sc*sky1*Δ and Cg*sky1*Δ mutants (**Figure 2**). Altogether, these results demonstrate a functional conservation of Sky1 among the examined species.

### *C. albicans SKY2* Is Required for Growth on Various Dipeptides as the Sole Nitrogen Source

**Figure 3:**
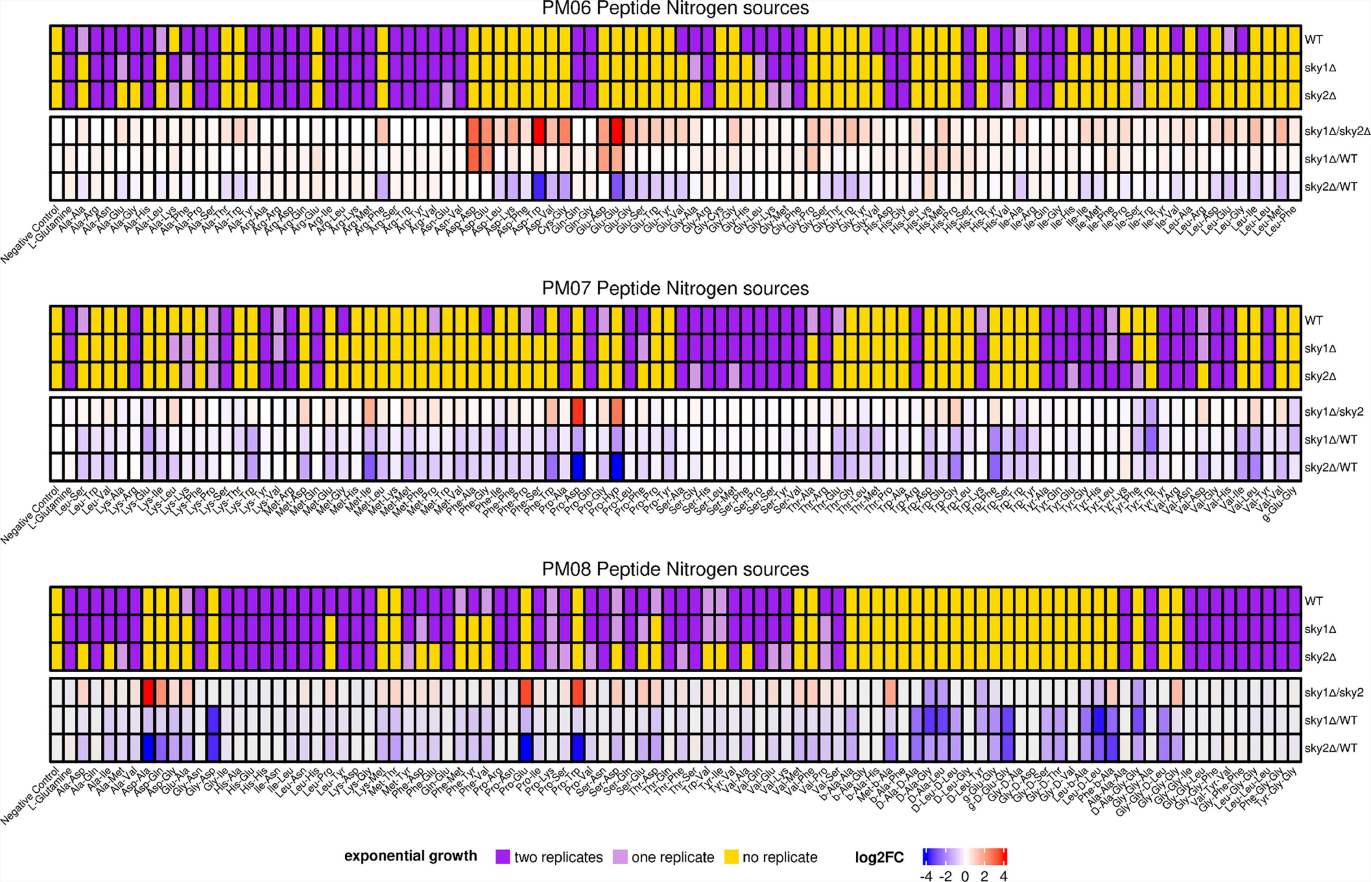
*C. albicans* Sky2 is required for growth on various dipeptides as the sole nitrogen source. The metabolic activities of the *C. albicans* SC5314 wild-type strain, Ca*sky1*Δ and Ca*sky2*Δ mutant strains A were measured kinetically every 15 min for 24 h at 30°C by utilizing Biolog™ phenotypic microarray plates for fungi. Upper panel: exponential growth phase according to the fitted logistic function in two, one or no replicate given for each tested nitrogen source. Lower panel: End point concentrations at 24 h were used to compute log_2_(fold changes). In the absence of an exponential growth characteristic in the investigated time frame, different end point concentrations and thus log_2_(fold changes) are due to different linear growth activities between the investigated strains.

Since we found divergences in the protein sequence and phenotypic differences between the two *C. albicans* SRPKs CaSky1 and CaSky2, we decided to apply a high-throughput phenotypic screen to further characterize their cellular roles. Given that the two independently generated Ca*sky1*Δ and Ca*sky2*Δ mutant strains showed the same phenotypes, we utilized only strain A of the respective mutants for this analysis. The Ca*sky1*Δ and Ca*sky2*Δ mutant strains were screened for metabolic activity on 904 different nutrients and supplements and compared to the metabolic activity of the wild-type strain SC5314. Overall, there were fewer and less pronounced phenotypic differences between the Ca*sky1*Δ mutant and the wild type compared to the Ca*sky2*Δ mutant vs. wild-type strain SC5314 (data not shown). Notably, the Ca*sky2*Δ mutant had impaired growth on multiple dipeptide combinations as the sole nitrogen source (**Figure 3**; **Supplementary Figure 2**). The Ca*sky2*Δ mutant was able to utilize only 104 out of the 268 tested dipeptides (38.8%), whereas the wild-type strain and the Ca*sky1*Δ mutant were able to utilize 146 (54.5%) and 125 (46.6%) dipeptides, respectively. Interestingly, the Ca*sky2*Δ mutant was as capable as the wild type in utilization of tripeptides, showing that the dipeptide growth defect is likely to be potentially specific (**Figure 3**; **Supplementary Figure 2**).

**Figure 4:**
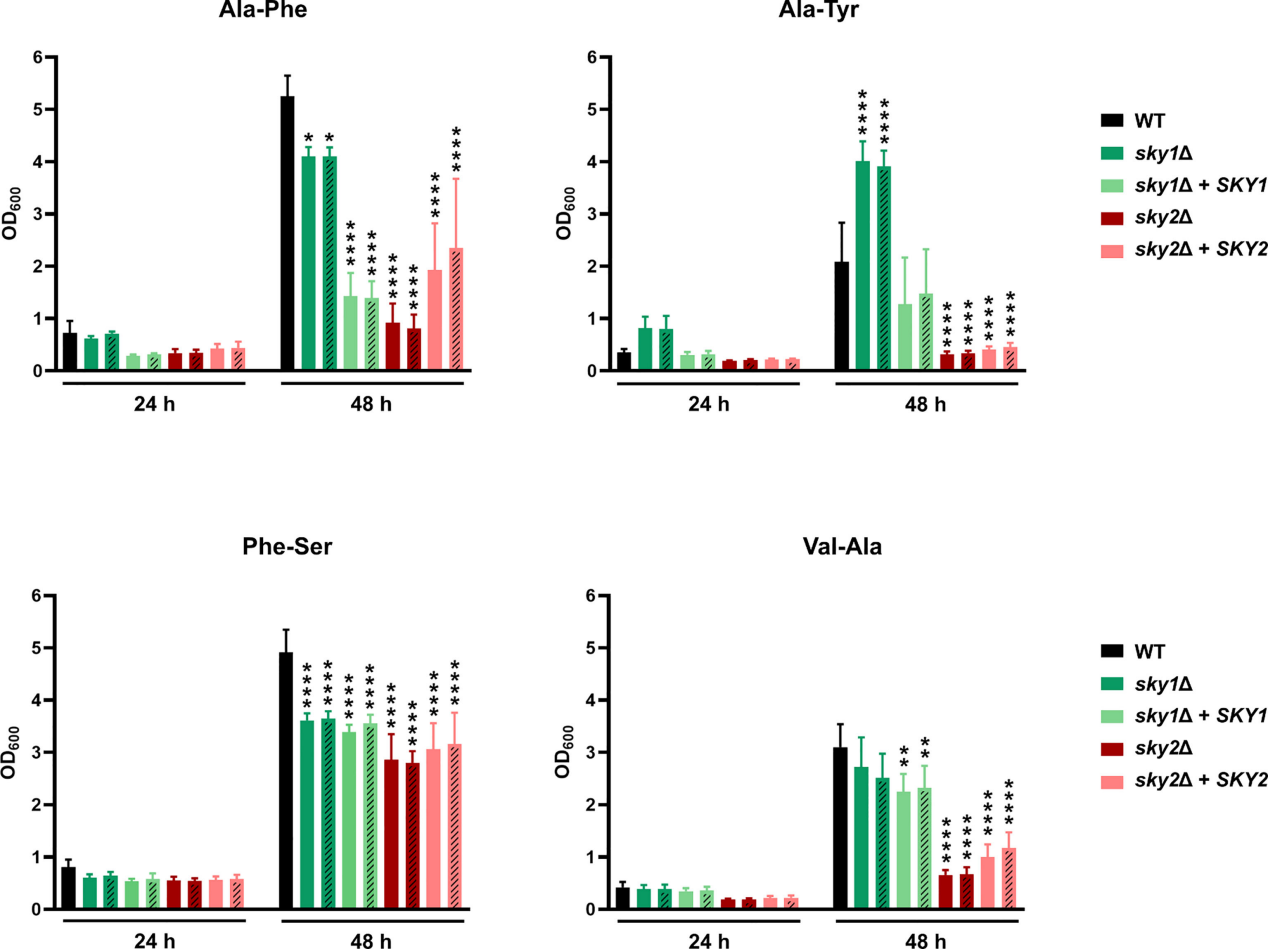
CaSky2 is important for utilization of selected dipeptides as the sole nitrogen source. YPD overnight cultures were adjusted to an optical density (OD_600_) of 0.01 in YCB medium containing 10 mM of the indicated dipeptide as a nitrogen source and incubated at 37°C. The OD_600_ was measured after 24 h and 48 h. Both independently generated series of mutants are displayed. The B strains are represented by dashes in the bars. The values shown are the calculated mean and standard deviation of three biological replicates. Growth differences of mutant strains to the wild-type strain SC5314 were analyzed by two-way ANOVA followed by Dunnett’s test (*, p < 0.05; **, p < 0.01; ****, p < 0.0001).

To validate the observations from the phenotypic screen we performed growth curve analyses using defined medium and added the B strains of the Ca*sky1*Δ and Ca*sky2*Δ mutants and the corresponding complemented strains. Based on the results of the phenotypic screening we selected as the sole nitrogen source several dipeptides on which the Ca*sky2*Δ mutant showed a growth defect (**Figure 3**): alanine-phenylalanine (Ala-Phe), alanine-tyrosine (Ala-Tyr) and valine-alanine (Val-Ala). Growth curve analyses confirmed that the Ca*sky2*Δ mutants exhibit significantly reduced growth compared to the wild-type strain and the Ca*sky1*Δ mutants on these particular dipeptides (**Figure 4**). Interestingly, Ca*sky1*Δ grew better on Ala-Tyr as the sole nitrogen source compared to the wild-type strain (**Figure 4**). We also tested growth on phenylalanine-serine (Phe-Ser), a dipeptide where both Ca*sky1*Δ and Ca*sky2*Δ mutants had impaired growth in the phenotypic screen. A similar defect was observed in the growth curve assay (**Figure 4**). Further, both Ca*sky1*Δ and Ca*sky2*Δ mutants showed reduced growth on Phe-Ser compared to the wild type, with Ca*sky2*Δ mutant strains growing significantly worse than the Ca*sky1*Δ mutants. The *SKY2* complemented strains either partially rescued the Ca*sky2*Δ mutant phenotype (Ala-Phe, Val-Ala) or exhibited growth comparable to the Ca*sky2*Δ mutant (Ala-Tyr, Phe-Ser). Both complemented strains were generated by re-introduction of allele A of *SKY2*. Comparison of the two *SKY2* alleles revealed some differences in their sequences, which prompted us to test the growth of the heterozygous *SKY2/sky2* mutant strains in which either allele A or allele B was still present on the selected dipeptides. Both heterozygous mutants showed comparable growth to the homozygous Ca*sky2*Δ mutant strains, indicating a gene dosage effect in which one *SKY2* allele is not sufficient to complement the phenotype of the wild-type strain (**Supplementary Figure 3**). Taken together, these results confirm the findings from the phenotypic screen that *C. albicans* Sky2 is required for assimilation of dipeptides as a nitrogen source.

### Phosphoproteome Analysis Reveals a Distinct Set of Potential Protein Targets for *C. albicans* Sky1 and Sky2 Protein Kinases

**Figure 5:**
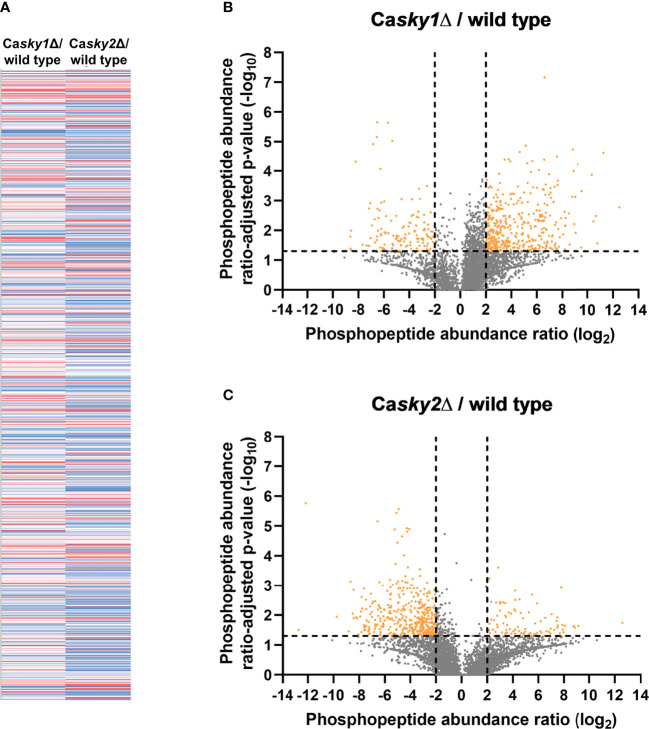
Phosphoproteome analysis revealed a distinct set of putative substrates for *C. albicans* Sky1 and Sky2. YPD overnight cultures were adjusted to an optical density (OD_600_) of 0.1 in YPD medium and incubated at 37°C for 4 h. Cells were harvested, lysed and proteins were tryptically digested, followed by phosphopeptide enrichment based on TiO2/ZrO2 resin (a non-enriched fraction served as reference). Three biological replicates were used per strain. **(A)** Heat map of all identified phosphopeptides (n=7243) by comparison of the fold change between *sky1*Δ versus wild type (left) and *sky2*Δ versus wild type (right) colored as follows: 10% quantile (blue), 50% quantile (white) and 90% quantile (red). **(B, C)** Volcano plots comparing the phosphopeptide abundance log2ratios (X axis) and the ratio-adjusted p-values as negative decade logarithm (Y axis) of all identified phosphopeptides for *sky1*Δ versus wild type **(B)** and *sky2*Δ versus wild type **(C)**. Highly significant changes are indicated as log2ratios < -2 and > 2 (4-fold change) and –log10 p-values > 1.3 (ratio adjusted p-value < 0.05).

We next performed a phosphoproteome analysis to identify the potential substrates responsible for the functional differences between CaSky1 and CaSky2. In brief, we grew the strains in YPD medium, extracted the proteins, and performed an LC-MS/MS analysis of tryptic peptides. TiO_2_/ZrO_2_-mediated phosphopeptide enrichment facilitated the identification and quantification of phosphopeptides with serine, threonine, and/or tyrosine phosphorylation. Phosphopeptide abundances were corrected against the corresponding master protein abundances (obtained from the non-enriched fraction) to enable a site-specific quantification. With this approach, we were able to identify 3946 proteins, 1663 phosphoproteins, 7243 phosphopeptides and 7727 phosphosites (82.05% serine, 16.57% threonine, 1.38% tyrosine) in total. Comparison of the fold change between the Ca*sky1*Δ mutant versus wild type and the Ca*sky2*Δ mutant versus wild type of all 3946 identified proteins revealed large differences in their quantile distributions (data not shown). Further, we identified 28 proteins significantly more abundant (log_2_ ratios > 2) and 31 proteins less abundant (log_2_ ratios < -2) in the Ca*sky1*Δ mutant versus wild type. On the other hand, we identified 31 proteins significantly more abundant (log_2_ ratios > 2) and 142 proteins less abundant (log_2_ ratios < -2) in the Ca*sky2*Δ mutant versus wild type, revealing differences between CaSky1 and CaSky2. The differences become especially clear on the phosphopeptide level when comparing the quantile distribution of all identified phosphopeptides (n = 7243) (**Figure 5A**) and the phosphopeptide abundance log_2_ ratios of the Ca*sky1*Δ mutant vs. wild type and the Ca*sky2*Δ mutant vs. wild type (**Figures 5B, C**). For the Ca*sky1*Δ mutant we identified 268 phosphopeptides assigned to 224 phosphoproteins with significantly altered abundance (175 more abundant (log_2_ ratios > 2), 49 less abundant (log_2_ ratios < -2) compared to the wild-type strain SC5314 (**Figure 5B**; **Supplementary Table 2**). In contrast, we identified 237 phosphopeptides assigned to 210 phosphoproteins significantly altered in abundance (23 more abundant (log_2_ ratios > 2), 187 less abundant (log_2_ ratios < -2)) in the Ca*sky2*Δ mutant compared to the wild-type strain (**Figure 5C**; **Supplementary Table 3**).

**Figure 6:**
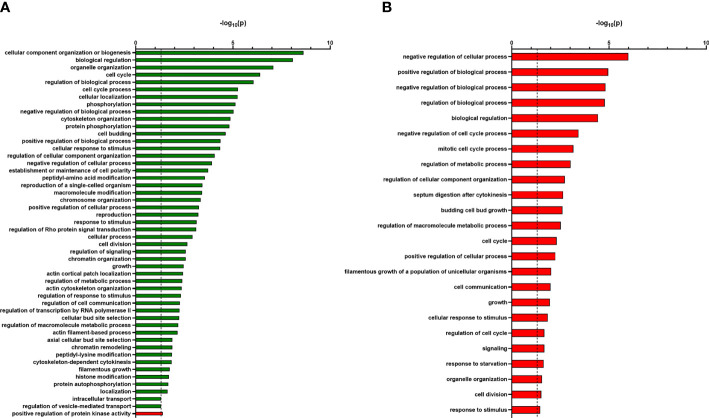
GO term analysis shows a different pattern of biological processes of the less and more abundant identified phosphoproteins of Ca*sky1*Δ versus wild type and Ca*sky2*Δ versus wild type. **(A, B)** GO terms of all identified phosphoproteins that were significantly more (green bars) or less abundant (red bars) in the Ca*sky1*Δ mutant compared to the wild-type strain SC5314 **(A)** and in the Ca*sky2*Δ mutant compared to the wild-type strain SC5314 **(B)** were analyzed using the GO term finder (CGD) and ‘process’ as a query and then using the Revigo tool (http://revigo.irb.hr/). Only significant GO terms defined by -log_10_(0.05) are shown (indicated by dashed line at 1.3 on X axis).

To find out in which biological processes the identified proteins are involved, we applied an in-depth GO term analysis. All identified phosphoproteins that were significantly more abundant in the Ca*sky1*Δ mutant compared to the wild-type strain SC5314 belong to several different GO terms like cellular component organization, cell cycle processes, cytoskeleton organization, cellular response to stimulus and regulation of transcription by RNA polymerase II (**Figure 6A**). For the identified 49 significantly less abundant phosphoproteins in the Ca*sky1*Δ mutant compared to the wild type, positive regulation of protein kinase activity was the only significant (-log_10_ p-values > 1.3) GO term that appeared in our analysis (**Figure 6A**). Although we could not identify any significant GO term for the 23 more abundant phosphoproteins in the Ca*sky2*Δ mutant compared to the wild type, 22 GO terms were identified for the 187 significantly less abundant phosphoproteins in the Ca*sky2*Δ mutant compared to the wild type, e.g. regulation of biological and cellular processes, regulation of metabolic processes, growth and response to starvation (**Figure 6B**).

**Figure 7:**
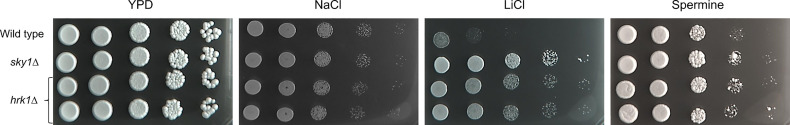
Deletion of *HRK1* confers resistance to high salt and toxic spermine levels. YPD overnight cultures of the strains were adjusted to an optical density (OD_600_) of 1.0. For *hrk1*Δ two independently generated mutants were tested. Serial 10-fold dilutions were spotted on YPD agar plates containing the indicated stressor and incubated for 2 days at 37°C. Plates contained 1.8 M NaCl, 0.3 M LiCl or 12.8 mM spermine.

Of the 49 less abundant phosphoproteins identified in the Ca*sky1*Δ mutant compared to the wild type, Hrk1 was the only protein kinase, with > 63 times (log_4_ fold change) lower phosphopeptide abundance and no detected phosphorylation at S81 and S95 (**Supplementary Table 2**). Since *HRK1* encodes an uncharacterized protein kinase to which we had available homozygous mutants (independently derived mutants A and B), we tested their resistance to osmotic stress and toxic polyamine concentrations. Interestingly, *hrk1*Δ mutants exhibited increased resistance to high LiCl and spermine concentrations, similar to Ca*sky1*Δ mutants, suggesting that Hrk1 is either a direct target of CaSky1 or an indirect target acting in the same pathway (**Figure 7**).

Among the 187 phosphoproteins that were significantly less abundant in the Ca*sky2*Δ mutant compared to the wild type, the transcription factors Fcr1 and Sfl1 as well as several protein kinases (Cst20, Gin4, Hsl1, Kic1, Ptk2, Sak1, Sat4, Swe1 and orf19.846) were identified as potential Sky2 targets. This suggests that CaSky2 is either part of a regulatory protein kinase network or that deletion of *SKY2* affects several pathways in the cell. Another potential target was the di- and tripeptide transporter Ptr22. The phosphopeptides were 70–85 times (log_4_ fold change) less abundant and no phosphorylation at S2, T3, and S39 was detected in the Ca*sky2*Δ mutant compared to the wild-type strain (**Supplementary Table 3**). We found this particularly interesting since Ptr22 and Ptr2 are the only known dipeptide transporters in *C. albicans*, with Ptr22 having a broader substrate spectrum than Ptr2 (Dunkel et al., 2013). Thus, in addition to altered phosphorylation, the observed strongly decreased abundance of Ptr22 may also explain the growth defect of the Casky2Δ mutants on dipeptides as the sole nitrogen source (**Figures 3**, **4**).

### *PTR22* Overexpression Overcomes the Growth Defect of the Ca*sky2*Δ Mutants on Dipeptides as the Sole Nitrogen Source

**Figure 8:**
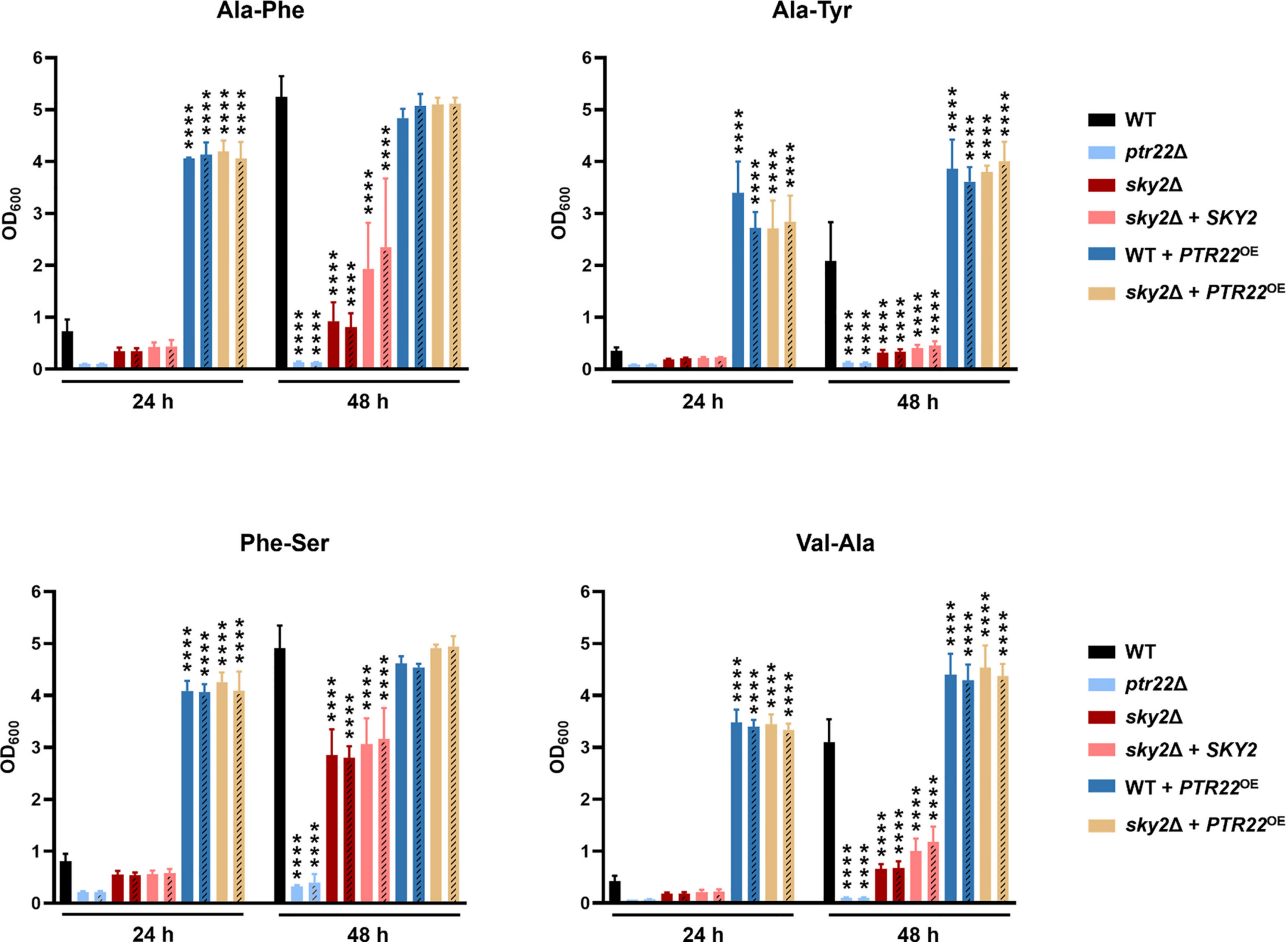
*PTR22* overexpression rescues the Ca*sky2*Δ mutant phenotype. YPD overnight cultures were adjusted to an optical density (OD_600_) of 0.01 in YCB medium containing 10 mM of the indicated dipeptide as a nitrogen source and incubated at 37°C. The OD_600_ was measured after 24 h and 48 h. The values shown are the calculated mean and standard deviation of three biological replicates. The independently generated B strains of each mutant are represented by dashes in the bars. Growth differences of mutant strains to the wild-type strain SC5314 were analyzed by two-way ANOVA followed by Dunnett’s test (*, p < 0.05; **, p < 0.01; ****, p < 0.0001). Data for the wild type, *sky2*Δ mutants, and *SKY2* complemented strains are the same as in **Figure 4**, since all strains shown in the two figures were tested in parallel.

To test whether Sky2 regulates dipeptide utilization *via* the di- and tripeptide transporter Ptr22, we generated strains overexpressing *PTR22* in the wild-type strain SC5314 and in the Ca*sky2*Δ mutant background and monitored their growth on the dipeptides Ala-Phe, Ala-Tyr, Phe-Ser, and Val-Ala as the sole nitrogen source. We also included two independently generated *ptr22*Δ mutants (strain A and B) as control, shown to have a growth defect on dipeptides (Dunkel et al., 2013). As expected, the two strains lacking *PTR22* were unable to grow on the tested dipeptides (**Figure 8**). Overexpression of *PTR22* under control of the constitutive *ADH1* promoter rescued the growth defect of the Ca*sky2*Δ mutants and significantly improved the growth of the wild-type strain on all dipeptides tested (**Figure 8**).

**Figure 9:**
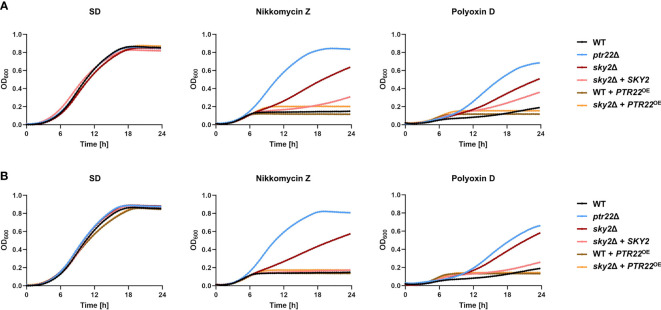
Strains lacking *SKY2* showed resistance to the antibiotics Nikkomycin Z and Polyoxin D. YPD overnight cultures were adjusted to an optical density (OD_600_) of 0.02 in SD medium and SD medium supplement with either 140 µg/ml Nikkomycin Z or 130 µg/ml Polyoxin D and incubated at 37°C for 24 h. The growth (OD_600_) was determined every 20 min at an optical density of 600 nm. Shown is the mean of each strain calculated from three technical replicates. For a better overview, the standard deviation is not shown. **(A)** The growth of the wild-type strain SC5314 and the A mutant strains is shown. **(B)** The growth of the wild-type strain SC5314 and the B mutant strains is shown.

It has been shown that *C. albicans* strains with defective uptake of di- and tripeptides are resistant to nikkomycins, polyoxins, and bacilysin, nucleoside antifungal agents that act as competitive inhibitors of chitin biosynthesis (Mehta et al., 1984; Payne and Shallow, 1985). Nikkomycin Z is in phase 2 clinical trial for treatment of coccidioidomycosis in humans (Nix et al., 2009) and we tested the susceptibility of the wild type and the mutant strains to this antifungal as well as to Polyoxin D. While these antifungals significantly impaired the growth of the wild-type strain, the *ptr22*Δ mutants were only slightly affected (**Figure 9**). Strains lacking *SKY2* exhibited resistance to both antifungals, but at a lower level than the *ptr22*Δ mutant strains. Growth of strains overexpressing *PTR22* in the wild type or in the Ca*sky2*Δ mutant background was completely inhibited by both antifungals (**Figure 9**). These results show that Ptr22 mediates the uptake of Nikkomycin Z or Polyoxin D, a function that is regulated in part by CaSky2.

## Discussion

The subfamily of SR protein kinases, defined by preferential phosphorylation of mRNA-binding proteins that contain SR/RS-enriched sequences, is highly conserved from yeasts to humans (Giannakouros et al., 2011). While the function of SRPK has been described in detail in the model yeasts *S. cerevisiae* and *S. pombe* where they control fundamental cellular processes such as mRNA processing and mitosis, their roles in human pathogenic fungi have not been previously investigated. This is the first work that examines their function in pathogenic *Candida* spp., specifically the SR protein kinases Sky1 and Sky2 in *C. albicans* and Sky1 in *C. glabrata*. Both CgSky1 and CaSky1 appear to have similar functions to the sole SRPK in *S. cerevisiae* ScSky1, whereas CaSky2 has both an expanded and diverged repertoire of target proteins, such as proteins involved in nutrient utilization.

While both model yeasts *S. cerevisiae* and *S. pombe* have a single SRPK1 homolog, SRPK gene expansions have been reported for filamentous fungi like *Aspergillus nidulans* (seven paralogous genes), *Neurospora crassa* (five), and several dermatophytes (18 to 34) (Martinez et al., 2012; De Souza et al., 2013). It is speculated that the response to a broad range of environmental conditions and the higher number of introns per gene in filamentous fungi has led to an increased importance of RNA processing, resulting in such expansion (De Souza et al., 2013). Pathogenic *Candida* spp. are also confronted with rapidly changing and diverse host environments. However, alternative splicing in *Candida* spp. likely plays only a minor role in host adaptation, well reflected by the low frequency of introns in *Candida* spp. genomes compared to other pathogenic fungi (Sieber et al., 2018). Furthermore, the duplication of SRPK genes seems to have occurred independently at different time points in the evolution of different taxa, since SRPKs lack a prominent one-to-one correspondence between the sequences, as proposed by (Giannakouros et al., 2011). The same authors reported the observation concerning the ‘spacer region’ that is characteristic for the SRPK family. This ‘spacer region’ is highly diverse in sequence length, and possibly in function, as the spacer seems to be unique for each SRPK family member. As illustrated in **Figure 1**, there is certain sequence diversity in the middle part of the kinase domain between the different homologs. However, the CaSky2 protein has three unique insertions of amino acid strings scattered in the kinase domain, indicative of possible functional differentiation. Indeed, *C. albicans* and *C. glabrata SKY1* deletion strains shared the *S. cerevisiae sky1*Δ resistance to high salt and toxic polyamine concentrations. A subsequent high-throughput phenotypic screen confirmed the substantial phenotypic differences between CaSky1 and CaSky2, supporting the notion of their divergent cellular functions. Whether these differences are due to the expanded ‘spacer regions’ of CaSky2 would require further investigation.

The main feature of human SRPKs is that they catalyze the phosphorylation of proteins enriched in serine/arginine recognition motifs, which earned them the name SR proteins (Zahler et al., 1992). In *S. cerevisiae* the confirmed protein targets of the SRPK1 homolog Sky1 also have multiple SR/RS sites, but those typically are randomly distributed throughout the protein and therefore referred to as SR-like. For example, ScSky1 phosphorylates the SR-like RNA-binding protein Npl3, which possesses eight SR/RS sites dispersed within the C-terminus of the protein (Siebel et al., 1999; Gilbert et al., 2001). Similarly, many of the direct or indirect target proteins of CaSky1 and CaSky2 identified in this study contain multiple randomly dispersed SR/RS sites. For example, the two proteins orf19.2459 or orf19.5051, which are among the 19 common potential substrates of CaSky1 and CaSky2, contain 20 and 16 SR/RS sites, respectively. Interestingly, the *C. albicans* ortholog of the *S. cerevisiae* Sky1 direct target ScNpl3, CaNpl3, was not among the potential phosphorylation targets of either CaSky1 or CaSky2. This is likely due to the substantially shorter protein length and the presence of only three SR/RS sites of CaNpl3 compared to the longer ScNpl3 protein that contains eight SR/RS sites. Thus, although direct evidence that CaSky1 and CaSky2 can phosphorylate proteins is still lacking, our data suggests that both are functional SR-like protein kinases.

Of the potential phosphorylation targets for both CaSky1 and CaSky2, we examined Hrk1, a protein kinase with a predicted role in cellular ion homeostasis. Deletion of *HRK1* resulted in the same resistance to LiCl and spermine as the deletion of Ca*SKY1*, suggesting either Hrk1 is a direct target of Sky1 or both are part of the same pathway. In addition to its role in regulation of ion homeostasis, CaSky1, similar to ScSky1, is potentially involved in RNA metabolism, as the putative mRNA export protein Elf1 was another prominent potential target of CaSky1. The functional relationship between Hrk1 and CaSky1 and the role of CaSky1 in mRNA metabolism requires further investigation. *C. glabrata* Sky1 also seems to exert similar cellular functions as ScSky1 and CaSky1. Thus, at least in these three species, Sky1 appears to regulate ion and possibly mRNA homeostasis.

Our analysis revealed substantial functional differences between CaSky1 and CaSky2, with CaSky2 having over 200 possible phosphorylation targets and distinct phenotypes. For instance, we identified several potential CaSky2 target proteins that are involved in metabolic processes, such as nutrient uptake, e.g. the di- and tripeptide transporter Ptr22. We found this particularly interesting, since strains lacking *SKY2* were defective for growth on multiple dipeptides and the cells were more resistant to the nucleoside antifungals Nikkomycin Z and Polyoxin D, both of which are most likely taken up *via* Ptr22 since *ptr22*Δ mutants failed to grow on dipeptides as the sole nitrogen source and were not susceptible to the tested antifungals. Furthermore, overexpression of *PTR22* in the *C. albicans* Ca*sky2*Δ mutants completely reversed the growth defect on dipeptides as the sole nitrogen source. Based on these findings, we hypothesize that Ptr22 phosphorylation is required for protein processing and/or its function.

We noted that the reduced growth of the Ca*sky2*Δ mutants on dipeptides and their resistance to Nikkomycin Z and Polyoxin D were not as prominent as the corresponding phenotypes of the ptr22Δ mutants. The intermediate phenotypes of Ca*sky2*Δ mutant cells were rescued by overexpression of *PTR22*, suggesting that CaSky2 could regulate Ptr22 stability. Such example is found in the *C. albicans* serine/threonine protein kinase Npr1, which promotes the transport-competent conformation of the ammonium transporter Mep2 (Neuhäuser et al., 2011). It is also feasible that CaSky2 is part of a protein complex that regulates Ptr22 function. Another possibility is that the deletion of *SKY2* alters the expression of other peptide transporters. The *C. albicans* genome encodes eight oligopeptide transporters (*OPT1*–*OPT8*), of which OPT1–5 have been identified as the major oligopeptide transporters that differ in their substrate preferences (Reuss and Morschhäuser, 2006). An ability to take up dipeptides has been shown for Opt1 and thus we cannot exclude the possibility for a low capacity/specificity transport *via* the OPTs. Indeed, qRT-PCR analysis of the Ca*sky2*Δ mutants grown on selected dipeptides resulted in significantly induced expression of *OPT2* and *OPT3* compared to the wild-type strain (data not shown). Whether Opt2 and Opt3 are involved in uptake of dipeptides following Ptr22 dysfunction needs to be examined further.

Among the most potential phosphorylation targets for CaSky2 were the SR/RS-enriched transcription factors Fcr1, a zinc cluster transcription factor and negative regulator of fluconazole, ketoconazole and brefeldin A resistance, and Sfl1, which is involved in the negative regulation of morphogenesis, flocculation, and virulence (Bauer and Wendland, 2007; Li et al., 2007; Shen et al., 2007). In addition, phosphorylation at specific phosphorylation sites of multiple protein kinases could not be identified in the Ca*sky2*Δ mutant compared to the SC5314 control strain, including Cst20, Gin4, Hsl1, Kic1, Ptk2, Sak1, Sat4, Swe1 and orf19.846. The Snf1-activating protein kinase Sak1 is a key regulator of metabolic adaptation and *in vivo* fitness in *C. albicans* (Ramírez-Zavala et al., 2017). Whether CaSky2 is involved in regulation of resistance to antifungals, morphogenesis or carbon metabolism directly or as a part of signalling cascade requires further investigation.

Altogether, this is the first study that focuses on the function of SRPKs in human-pathogenic fungi. Herein, we show that the SR-like kinase Sky1 is involved in the regulation of ion homeostasis in *C. albicans* and *C. glabrata*, as its ortholog in *S. cerevisiae*. The CTG clade-specific kinase Sky2 plays a role in utilization of dipeptides in *C. albicans*, a novelty for the function of SRPKs.

## Data Availability Statement

The datasets presented in this study can be found in online repositories. The names of the repository/repositories and accession number(s) can be found in the article/**Supplementary Material**.

## Author Contributions

PB, FG, TK, BR-Z, and OK designed and performed the wet lab experiments. PB, LW, MM, and SS performed in silico analyses. PB, FG, LW, TK, MM, and SS analyzed the data. PB, FG, LW, and MM designed the figures. GP, AB, JM, and SV discussed the results and supervised the project. PB, LW, and SV wrote the manuscript in consultation with TK, BR-Z, SS, and JM. All authors contributed to the article and approved the submitted version.

## Funding

This study was funded by the German Ministry for Education and Science in the program Unternehmen Region (BMBF 03Z22JN11) (to SV) and the German Research Foundation (DFG) through the TRR 124 FungiNet, “Pathogenic fungi and their human host: Networks of Interaction,” DFG project number 210879364, project C2 (to SV and JM), B5 (to GP), project INF (to GP), and project Z2 (to OK).

## Conflict of Interest

The authors declare that the research was conducted in the absence of any commercial or financial relationships that could be construed as a potential conflict of interest.

## Publisher’s Note

All claims expressed in this article are solely those of the authors and do not necessarily represent those of their affiliated organizations, or those of the publisher, the editors and the reviewers. Any product that may be evaluated in this article, or claim that may be made by its manufacturer, is not guaranteed or endorsed by the publisher.
