# Supplementary material for: Candida albicans SR-Like Protein Kinases Regulate Different Cellular Processes: Sky1 Is Involved in Control of Ion Homeostasis, While Sky2 Is Important for Dipeptide Utilization
Source: Front Cell Infect Microbiol. 2022 May 6;12:850531. doi: 10.3389/fcimb.2022.850531 (PMC9121809; doi:10.3389/fcimb.2022.850531)
Supplement: Supplementary file 1 [file DataSheet_1.docx]

*Candida albicans* SR-like protein kinases regulate different cellular processes: Sky1 is involved in regulation of ion homeostasis, while Sky2 is important for dipeptide utilization

Philipp Brandt ^1^, Franziska Gerwien ^1^, Lysett Wagner ^1^, Thomas Krüger ^2^, Bernardo Ramírez-Zavala ^3^, Mohammad H. Mirhakkak ^4^, Sascha Schäuble ^4^, Olaf Kniemeyer ^2^, Gianni Panagiotou ^4,5^, Axel A. Brakhage ^2^, Joachim Morschhäuser ^3^ and Slavena Vylkova ^1^ *

**Supplementary figures**

**Supplementary Figure 1**

**
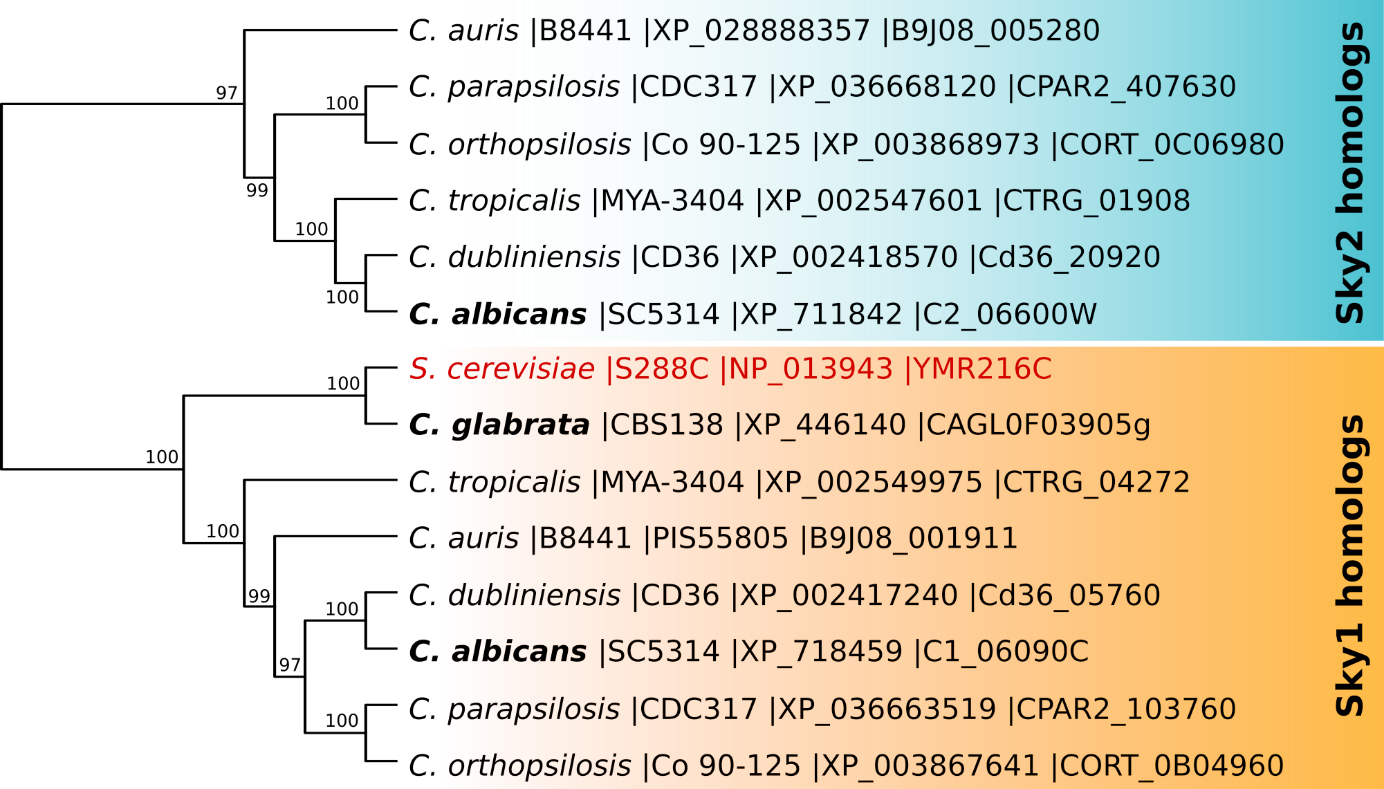
**

**Supplementary Figure 1. BLASTp of the human SRPK1 protein (NCBI Reference Sequence: NP_003128.3).** Except for *C. glabrata* the most common pathogenic *Candida* spp. possess two SRPK homologs, all having a high level of homology to *S. cerevisiae* Sky1 protein kinase. [species |strain |GenBank number |model name on CGD]

**Supplementary Figure 2**


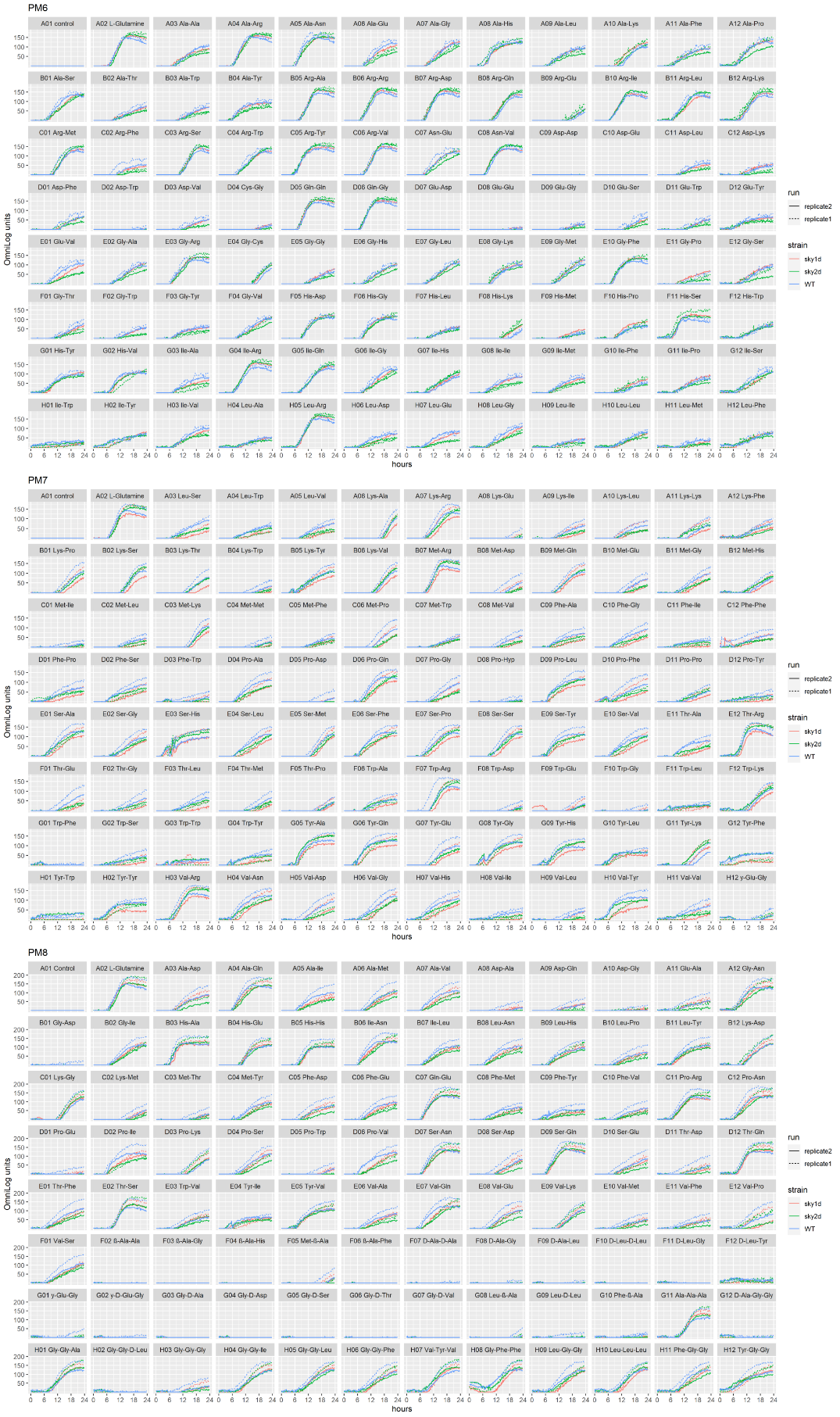


**Supplementary Figure 2. *C. albicans* Sky2 is required for utilization of dipeptides as the sole nitrogen source.** The metabolic activities of of two biological replicates of the *C. albicans* SC5314 wild-type strain, Ca*sky1*∆ and Ca*sky2*∆ mutant strains A were measured kinetically every 15 min for 24 h at 30°C by utilizing Biolog™ phenotypic microarray plates for fungi. The metabolic acitives are shown after subtracting the negative control (well A1 of each plate).

**Supplementary Figure 3**


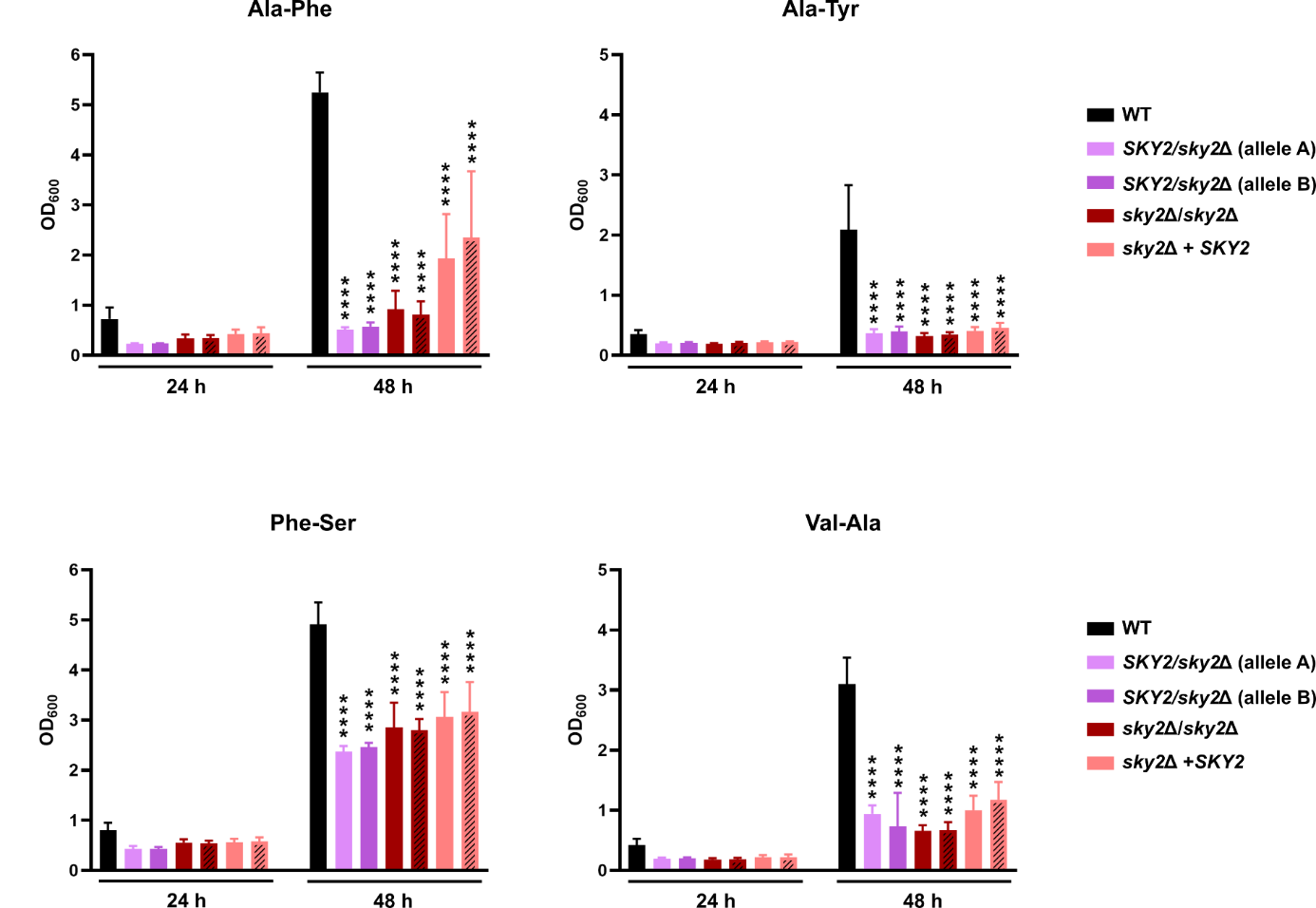


**Supplementary Figure 3. Heterozygous *SKY2/sky2*Δ mutant strains show a similar growth defect on dipeptides as the sole nitrogen source as the homozygous Ca*sky2*Δ mutant strains.** YPD overnight cultures were adjusted to an optical density (OD_600_) of 0.01 in YCB medium containing 10 mM of the indicated dipeptide as a nitrogen source and incubated at 37°C. The OD_600_ was measured after 24 h and 48 h. One heterozygous *SKY2/sky2*Δ mutant with a remaining allele A and one with a remaining allele B were tested. The independently generated B strains of the homozygous Ca*sky2*Δ mutant and the *SKY2* complemented strain are shown by dashes in the bars. The values shown are the calculated mean and standard deviation of three biological replicates. Growth differences of mutant strains to the wild-type strain SC5314 were analyzed by two-way ANOVA followed by Dunnett’s test (*, p < 0.05; **, p < 0.01; ***, p < 0.001; ****, p < 0.0001).
